# Realising the potential of Natura 2000 to achieve EU conservation goals as 2020 approaches

**DOI:** 10.1038/s41598-019-52625-4

**Published:** 2019-11-06

**Authors:** Virgilio Hermoso, Alejandra Morán-Ordóñez, Stefano Canessa, Lluis Brotons

**Affiliations:** 1 Centre de Ciència i Tecnologia Forestal de Catalunya (CTFC), Crta. Sant Llorenç de Morunys, km 2, Solsona, 25280 Spain; 2 Australian Rivers Institute, Griffith University, Nathan, Qld 4111 Australia; 3 InForest JRU (CTFC-CREAF), Crta. Sant Llorenç de Morunys, km 2, Solsona, 25280 Spain; 4 Wildlife Health Ghent, Faculty of Veterinary Medicine, Ghent University, Merelbeke, B9820 Belgium; 5 CREAF, Cerdanyola del Vallés, 08193 Spain; 6 CSIC, Cerdanyola del Vallés, 08193 Spain

**Keywords:** Biodiversity, Conservation biology

## Abstract

In the last decades the EU has made substantial efforts implementing conservation strategies to halt biodiversity loss. However, little improvement has been reported. Given the proximity of the 2020 landmark set by the EU Biodiversity Strategy and the Convention for Biological Diversity, alternatives to reduce this conservation gap and prospect future strategies must be assessed urgently. Here, we explore how the current Natura 2000 could be used to enhance management of terrestrial and freshwater threatened vertebrates. We identified Natura 2000 sites to increase the coverage of threatened species as target species under two alternative scenarios: a policy-driven approach including only threatened vertebrates listed in the Directives; and a conservation-driven approach, including all the remaining threatened vertebrates. We show that representation of threatened vertebrates in Natura 2000 could be improved by updating lists of target species in less than 1% and 3% of sites in the policy-driven and conservation-driven scenarios, respectively. We highlight the strength of Natura 2000, with sites that complement each other and could contribute to achieving more ambitious conservation targets under future strategies. Prioritisation exercises like this could help realise the potential of this network and enhance the management of threatened species and improve current gaps.

## Introduction

In 2011 the European Commission adopted its Biodiversity Strategy aimed at ‘halting the loss of biodiversity and the degradation of ecosystem services in the EU by 2020’^1^ following up unmet targets set for 2010. To achieve this continental goal, the EU’s nature and biodiversity policy relies on the Birds and Habitats Directives (Directive 2009/147/EC, 92/43/EEC). The implementation of these Directives has led to the declaration of one of the world’s largest networks of protected areas (PAs), the Natura 2000 network, that currently represents the centrepiece of the EU Biodiversity Strategy. The network comprises more than 27,000 PAs and covers >1 million km^2^, 18.3% of the EU’s land surface and about 6% of its marine area^2^. The EU has also endorsed the Strategic Plan for Biodiversity of the Convention on Biological Diversity^3^ that shares conservation goals and deadline with the EU’s Biodiversity Strategy.

Although there is evidence of the positive impact of the conservation efforts carried out by the EU Member States over the last three decades in setting up and implementing the Natura 2000 network (e.g.)^4,5^, the overall improvement in the conservation status of species and habitats reported in the last European assessment^6^ is still far from the targets set for 2020 in the Biodiversity Strategy. In 2015 (last available report on conservation status of EU biodiversity) there were only 1–2% more species in favourable status than in 2007 (previous reporting period), while there is still a significant proportion of species whose condition continues to deteriorate^7^. Commonly mentioned factors behind the limited conservation impact of Natura 2000 are the insufficient budget to cover costs associated to the management of the network^8^, the lack of adequate planning when designing the network, overlooking aspects like connectivity or representativeness^9–12^, the persistence of conflicting interests and competing policies (see e.g.)^13^, and poor management of PAs, many of which still lack management plans and could be considered “paper parks”^14,15^. In 2012, only 30% of areas under the Birds Directive and 41% of areas under the Habitats Directives had implemented management plans. Developing management plans is the responsibility of Member States, and is critical for the adequate implementation of the Directives and the Natura 2000. The lack of these management plans constrains the capacity of the EU’s conservation efforts to achieve the 2020 targets, as no specific management is prescribed to ensure PAs are effectively managed or conservation objectives achieved.

Moreover, the estimates on conservation status reported in the EU’s biodiversity assessments give only a partial view of the overall condition of EU biodiversity. These estimates overlook the magnitude of the conservation problem, given the poor correspondence between the threatened status of species (as, for example, reported by the IUCN’s Red List^16^) and the species listed in the annexes of the Directives. For example^12^, reported that there are 29 terrestrial species of mammals, reptiles and amphibians endemic to the EU and threatened with extinction that are not included in the annexes, while 82% of the vertebrates listed are not considered threatened at the global level (IUCN). Similar issues have also been described for other comprehensively assessed taxa, such as some insects^17^, butterflies^18^ or dragonflies^19^. The achievement of the Biodiversity Strategy could be compromised by the low representation of threatened species in the mentioned annexes^19^, which adds to the weak improvement in the conservation status of listed species.

Recent studies have proposed solutions to improve the impact of conservation efforts within the Natura 2000 network. Alternatives range from increasing the budget devoted to managing the network^20,21^, to revisiting management plans and the lists of target species to be managed, and to designating additional protected areas to better cover threatened species^14,15^. These changes should be informed by adequate planning, to enhance the impact of conservation measures under limited budgets^22,23^. For example, conservation funds under the LIFE programme, the main financial tool of conservation action in the EU, have been increasing over time^22^. However,^23^ recommended that this increase needs to be accompanied by a more strategic planning of the distribution of these funds to ensure they are directed towards those species and habitats most in need. As suggested by^24^ current fixed lists of species used to define management priorities should be replaced with regular updated species lists, to match changes in scientific evidence on conservation needs (although see for potential issues)^25^. This adaptive approach, informed for example by changes in conservation status of species, could contribute to increasing our capacity to respond to changing conservation challenges derived from the effects of global change^14,23,26^. Finally, new protected areas might be needed to fill the coverage of threatened species^10,14^. However, expanding a network that is already large and potentially conflicting with other land uses^11,12,27^, will require adequate planning and political will.

In this study, we demonstrate how the use of conservation planning tools can help to address some of the above-mentioned challenges to EU biodiversity conservation. These tools help in the decision-making process by providing robust analyses on how to best achieve conservation goals at minimum socio-economic impact. We assess to what extent the coverage of threatened species can be improved within the current boundaries of the Natura 2000 sites, avoiding the designation of new protected areas. We identify Natura 2000 sites where local lists of target species could be updated to improve the EU-wide coverage of threatened species. The Natura 2000 network can cover not only species listed in the annexes of the Directives^27^, but also other non-listed species. For this reason, we tested two alternative scenarios: a policy-driven approach including only threatened vertebrates listed in the Directives; and a conservation-driven approach, including all the remaining threatened vertebrates. Our analysis seeks to maximize this “umbrella effect of Natura 2000” (*sensu*)^27^, by identifying priority Natura 2000 sites where it would also be possible to manage threatened species (especially non-listed) that currently are not necessarily the target of management actions.

## Results

A total 400 of the 1282 species of European terrestrial and freshwater vertebrates are listed in the annexes I and II of the Birds Directive and II and IV of the Habitats Directive. Of these, 113 species (28%) are threatened with extinction as assessed by the latest edition of the IUCN Red List^16^. Of the 882 remaining species of vertebrates not included in the annexes, a total of 322 (36%) are threatened. These numbers differed across taxonomic groups. For example, freshwater fish were the taxonomic group with both the largest number of threatened species (N = 156) and the lowest proportion of these covered by the Directives’ annexes (13%). Conversely, 81% of the 49 species of threatened birds were listed in the annexes I and II of the Birds Directive (Table 1).

**Table 1:** Summary of number of species included in each taxonomic group and IUCN’s status (numbers in bold) and listed in the annexes of the Birds (I and II) and Habitats (II and IV) Directives.

Taxa	IUCN status							Total threatened (CR, EN, VU, NT)	Total threatened in Directives
	CR	EN	VU	NT	LC	NE	DD		
Amphibians	**2**/0	**5**/1	**11**/4	**15**/6	**49**/26	**2**/0	**1**/0	33	11
Birds	**2**/1	**8**/7	**15**/11	**24**/21	**428**/173	**0**/0	**0**/0	49	40
Fish	**45/**7	**38**/6	**57**/6	**16**/2	**172**/27	**45**/0	**23**/1	156	21
Mammals	**2**/2	**5**/2	**14**/8	**19**/14	**125**/31	**10**/5	**11**/3	40	26
Reptiles	**6**/2	**12**/5	**8**/3	**18**/5	**79**/24	**12**/1	**3**/0	44	15
Total	**57**/12	**68**/21	**105**/32	**92**/48	**853**/277	**69**/6	**38**/4	322	113

Numbers based on the IUCN’s Red List 2019^28^. CR = Critically Endangered; EN = Endangered; VU = Vulnerable; NT = Near Threatened; LC = Least Concern; NE = Not evaluated; DD = Data deficient. Only species listed as CR, EN, VU and NT were considered threatened^29–31^.

**Figure 1:**
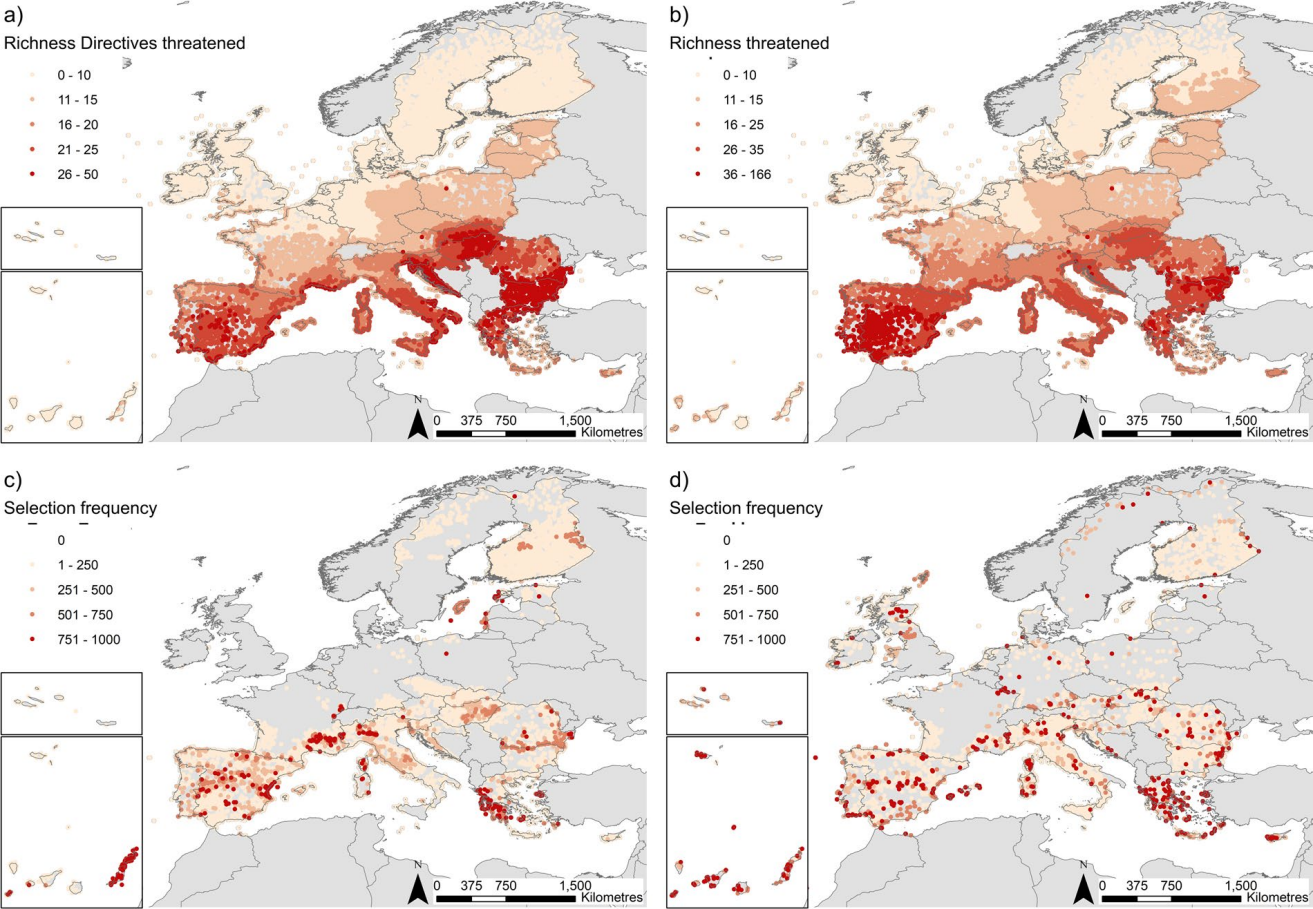
(**a**) Richness of threatened vertebrate species listed in the Habitats and Birds Directives and (**b**) all threatened vertebrates (IUCN listed), across the Natura 2000 network; c) selection frequency of each Natura 2000 site under policy-driven and d) conservation driven scenarios (including only threatened species listed in the Directives or all threatened species, respectively). The higher the selection frequency, the larger the contribution of the site to the management of threatened species, as sites are recursively selected regardless of the subset of species being tested in each of the 1000 bootstrap prioritisation analyses. Natura 2000 sites are represented by their centroid for mapping purposes.

There is a clear latitudinal gradient in the number of threatened species occurring within Natura 2000 sites, with southern countries hosting most of the threatened species (Fig. 1). This pattern is similar when looking at all threatened species, or just threatened species listed in the Directives. On the other hand, there was not a clear spatial pattern in the number of target species currently declared for Natura 2000 sites (Supplementary Fig. S1). Note that some Natura 2000 sites had no target species declared and were designated for protecting habitats in Annex I of the Habitats Directive.

The spatial prioritisation carried out in Marxan aimed to identify a minimum of 10 Natura 2000 sites (representation targets) where threatened species could be included as target species, under the two alternative scenarios (policy- and conservation-driven). These representation targets were achieved for all species under both scenarios. This could be done by updating the lists of target species on average in 231 (±4) and 845 (±5) Natura 2000 sites under the policy-driven and conservation-driven scenarios, respectively.

Natura 2000 sites in southern Europe (Spain, Portugal, Greece, southern France and northern Italy) and some of the islands in Macaronesia (especially the Canary Islands) appeared consistently in priority solutions regardless the particular pool of species being used across bootstraps and scenarios (Fig. 1c,d). Natura 2000 sites in southern Finland and countries in central and eastern Europe were also selected but with lower frequencies than the former, which might indicate a higher potential for replaceability among them. Despite similarities in the spatial pattern of selection frequency, there were more sites recursively selected in central and northern Europe when all threatened vertebrate species were considered (Fig. 1d). For example, some Natura 2000 sites in northern UK, Germany and the Czech Republic showed high selection frequency under this scenario, reflecting the occurrence in those sites of threatened species that are not listed under the Directives’ annexes. There was no relationship between the richness of threatened vertebrate species or the richness of threatened vertebrate species from the Directives’ annexes and the site selection frequency (Supplementary Fig. S2), which indicates that the probability of selecting a site was not strongly driven by the local richness of a Natura 2000, but the identity of the species occurring in them. The selection frequency of sites was sensitive to the size of the pool of additional species allowed in a given Natura 2000 site to contribute to the target achievement (species threshold): the larger the number of species allowed to be added as target species per site, the more concentrated was the selection frequency on a smaller number of sites, although with a similar distribution across thresholds (Supplementary Figs S3 and S4).

**Figure 2:**
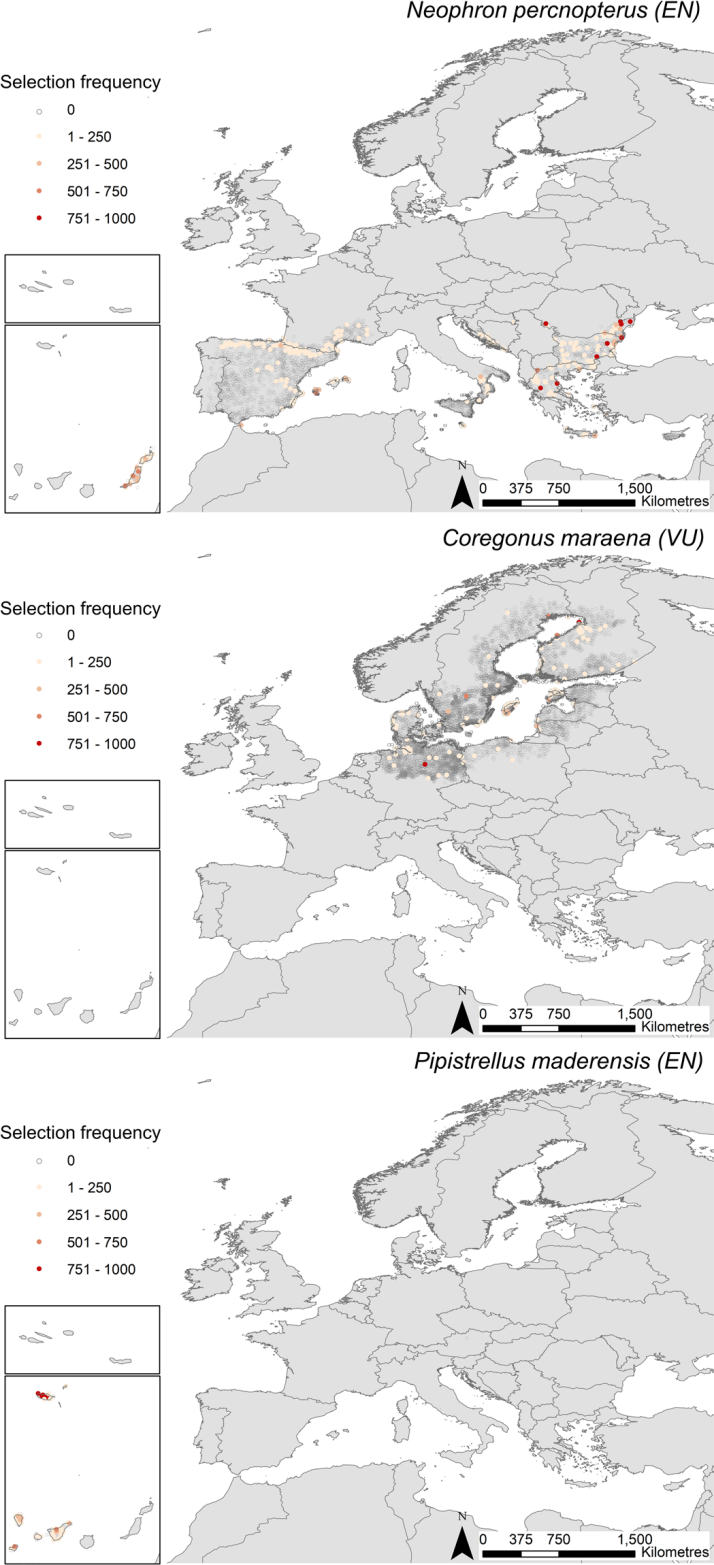
Selection frequency of three different species in Natura 2000 sites where they occur across 1000 bootstraps: the Egyptian vulture *Neophron percnopterus*, the maraena whitfish *Coregonus maraena*, and the Madeira Pipistrelle *Pipistrellus maderiensis*. High frequency represents Natura 2000 sites that were recursively selected for each species and then identified as priority sites where the species should be added to the current list of target species. Natura 2000 sites are represented by their centroid for mapping purposes.

Finally, the combination of the bootstrapping and the prioritisation approach rendered the selection frequency of each species at each Natura 2000 site. In this way we could also identify which Natura 2000 sites would be the most adequate for managing these additional species. For example, for medium-widely distributed species across Europe, such as the Egyptian vulture (*Neophron percnopterus*, EN) or the Maraena whitefish (*Coregonus maraena*, VU), there were a few priority sites selected across their whole distribution range, some of them with a high frequency (Fig. 2a,b). On the other hand, other species with limited distribution ranges, such as the Madeira Pipistrelle (*Pipistrellus maderensis*, VU) was selected in almost all Natura 2000 sites where it occurs with a high frequency (Fig. 2c). However, even in the case of these rare species, there were differences in the selection frequency of Natura 2000 sites where they occur (Fig. 2). Interestingly, the selection of sites for a given species tended to be more evenly distributed when only considering threatened vertebrate species listed in the Directives (policy-driven scenario) than when broadening the prioritisation to all threatened species (conservation-driven scenario). This happened mainly because of the consideration of additional threatened species endemic of northern regions, not listed in the annexes of the Directives (e.g., freshwater fish species of genus *Coregonus* only present in Northern UK).

## Discussion

Our study shows that threatened terrestrial and freshwater vertebrates could be represented more effectively within the current extent of the Natura 2000 network, with no additional designation of protected areas. We show that this could be done by modifying the lists of target species in a small proportion of the sites within the current network. By updating the lists of target species in less than 1% and 3% of Natura 2000 sites identified in the prioritisation exercise in policy-driven and conservation-driven scenarios, respectively, all threatened terrestrial and freshwater vertebrates could be declared as target species in at least 10 Natura 2000 sites. When prioritising new species additions to current lists of target species, we also accounted for management currently implemented in Natura 2000 sites, as a strategy to minimise the need for additional management efforts. We prioritised threatened species that could benefit from already existing management whenever possible. The recommendations that arise from this systematic planning exercise could be used for prioritising management on the ground to cover conservation needs for all species and habitats that face extinction risk. In this way, our approach could contribute to improving the management of threatened biodiversity in the EU and contribute to the achievement the continental goals committed in the EU’s Biodiversity Strategy, minimising its socio-economic impact, and potentially unlock conservation opportunities for all threatened species.

Improving the impact of conservation efforts in the EU requires, among other things, revising the management of species currently listed in the Directives’ annexes, for which little or no improvement has been reported in the last two decades (2007–2012)^6^. Moreover, given the large number of threatened species not actually covered by the Directives, halting loss of biodiversity in the EU requires moving beyond the fixed lists that currently guide conservation efforts to better cover species threatened with extinction^14,15,22^. The dynamism of populations and threats poses important challenges to conservation practitioners, who should adapt their efforts and priorities as conditions change, rather than adhering to rigid priorities. This is especially relevant in a context of global change, where the conservation status of species is expected to constantly change. On the other hand, managing all threatened species implies socio-economic (e.g., conflict with other land uses^27^) and political challenges (e.g., lack of political support^28^), that could be eased through adequate planning^10,11^, by better distributing limited conservation funds across species and protected areas (e.g.^23^). We have demonstrated that the Natura 2000 network already offers opportunities to cover the distribution of not only species listed on the annexes, but also other threatened vertebrates. Updating lists of target species per Natura 2000 site to cover even non-listed threatened species might be seen as out of the current policy coverage and then difficult to implement. However, the EU policy already includes mechanisms to add species of conservation concern to the lists of target species regardless of whether they are listed in the annexes of the Directives (see for example section 3.3 on the Standard Data Form of the Natura 2000 sites^32^).

Our findings do not imply that more protected areas would not benefit conservation^10^, especially when considering other taxonomic groups like invertebrates or plants not included in this study. Additional protected areas or management efforts of biodiversity outside Natura 2000 might be needed to cover those groups adequately. However, in general, the combination of high population density and pressure on productive land and the existence of a large network of protected areas in the EU, makes further designation of protected areas socially, economically, and politically costly^12^. In fact, the rate of new additions to Natura 2000 has slowed down in the last years^11^. Future efforts should focus on improving management of Natura 2000 sites whenever possible, to increase conservation outcomes and minimise the need for new protected areas. In cases, where species cannot be managed under the current Natura 2000, additional effort might be prescribed in new designated protected areas or outside them.

Our conservation planning approach seeks to minimise the potential socio-economic and political conflicts of the additional conservation effort needed at the EU level, by extending management to threatened species that co-occur with other species listed in the Directives that are already managed in extant protected areas. In this sense, our results do not imply changing the number, location or boundaries of protected areas but, whenever possible, revisiting the lists of target species to add a number of new species to be managed (always maintaining the ones that are already being managed). We tried to further minimise the economic impact of these new additions, by considering current management efforts being carried out on the ground for target species (using the habitats they occupy as indicator of current management efforts). So, priority was given to threatened species, listed or not in the annexes of the Directives depending on the planning scenario, that could co-benefit from these efforts and by limiting the number of new additions allowed on a single Natura 2000. Additional resources might still be necessary, even when the species added occupy similar habitats to the ones already being managed, to ensure protected areas effectively safeguard the persistence of populations.

Results from such prioritisation approaches could also inform the future development of management plans for all those Natura 2000 sites that still lack them^14^. However, translating continental priorities into regional or local management is not easy and it might be compromised by stakeholder reluctance to a top-down approach for defining conservation needs^11^, and by the need to balance biodiversity conservation targets against human priorities in each Member State or region^33^. The outputs of the prioritization exercise could, however, inform the development of action plans at continental scale. These action plans aim to identify and prioritise measures to restore populations of species across their range within the EU and help improve the conservation status of the species. There are very limited number of species with EU-wide action plans in place (3 species of the Habitats Directive and 70 bird species from the Birds Directive; see^34^ for an example) and results could then help identify the Natura 2000 sites where these measures would be more relevant at a continental scale.

The data we used to identify priority sites was based on the occurrence of the species within protected areas, derived from the Red List distribution data. We assumed that if the distribution range of a species overlapped a given Natura 2000 site, the species could potentially benefit from the management carried out in that site. However, the declaration of target species within a given site responds to population rather than occurrence criteria. Planning at the continental scale requires the use of standardised data across the EU to avoid biases in the spatial prioritisation towards areas with better data; however, population data is not available at continental scale for all threatened species. Despite its limitations, IUCN data has been used in similar conservation assessment exercises in Europe (see^35,36^) and elsewhere^37–39^. The distribution data used in the study conveys the extent of species occurrence^40^. Although it might overestimate the number of Natura 2000 sites where each species occurs, the bootstrap approach we followed, where only a subset of the occurrences for each species were used iteratively, could help reduce the effect of overestimated distributions. As we show, regardless of the particular subset of occurrences used, some Natura 2000 sites appeared recursively as priority in our solutions. Moreover, the selection frequency was independent of the local richness of Natura 2000 sites, which indicates that potential biases towards Natura 2000 sites with higher estimated richness due to the overlap of multiple species’ overestimated distributions was minimum in our results. The actual implementation of this method to design a conservation strategy at the EU scale would require species distribution data of higher spatial resolution, and will need further data about other threatened taxa for which distribution and environmental constraints are scarcely known (e.g. invertebrates).

Matching previous studies, the Natura 2000 sites that more recursively appeared in our solutions were mainly located in southern EU and Macaronesia (e.g.,^11,22^). These sites were not necessarily the richest in threatened species (Supplementary Fig. S2), but hold endemic species with restricted distribution ranges, such as some amphibians and reptiles^27^. Although the spatial patterns rendered by both planning scenarios were very similar, the consideration of all threatened vertebrates (conservation-driven scenario) broadened the selection of Natura 2000 sites towards northern EU (Fig. 1d). These results highlight two key messages for future EU conservation planning. First, the whole network is needed to cover all threatened vertebrates and therefore, to contribute to the achievement of the EU’s conservation commitments. Second, southern regions play a key role in maintaining a large proportion of the EU’s threatened biodiversity. Financial instruments such as the LIFE program must support conservation in these areas^22,23^ and share the responsibility of achieving continental conservation targets.

Although it might be too late to achieve the 2020 objectives, these ideas can contribute to the ongoing development of post-2020 biodiversity strategies. Our results show the great potential of the existing Natura 2000 network for the management of all threatened vertebrates in the EU. Adequate planning can help realise this potential, by prioritising where the additional conservation efforts should be allocated, minimising potential socio-economic and political conflicts.

## Methods

### Natura 2000 and species distribution data

**Figure 3:**
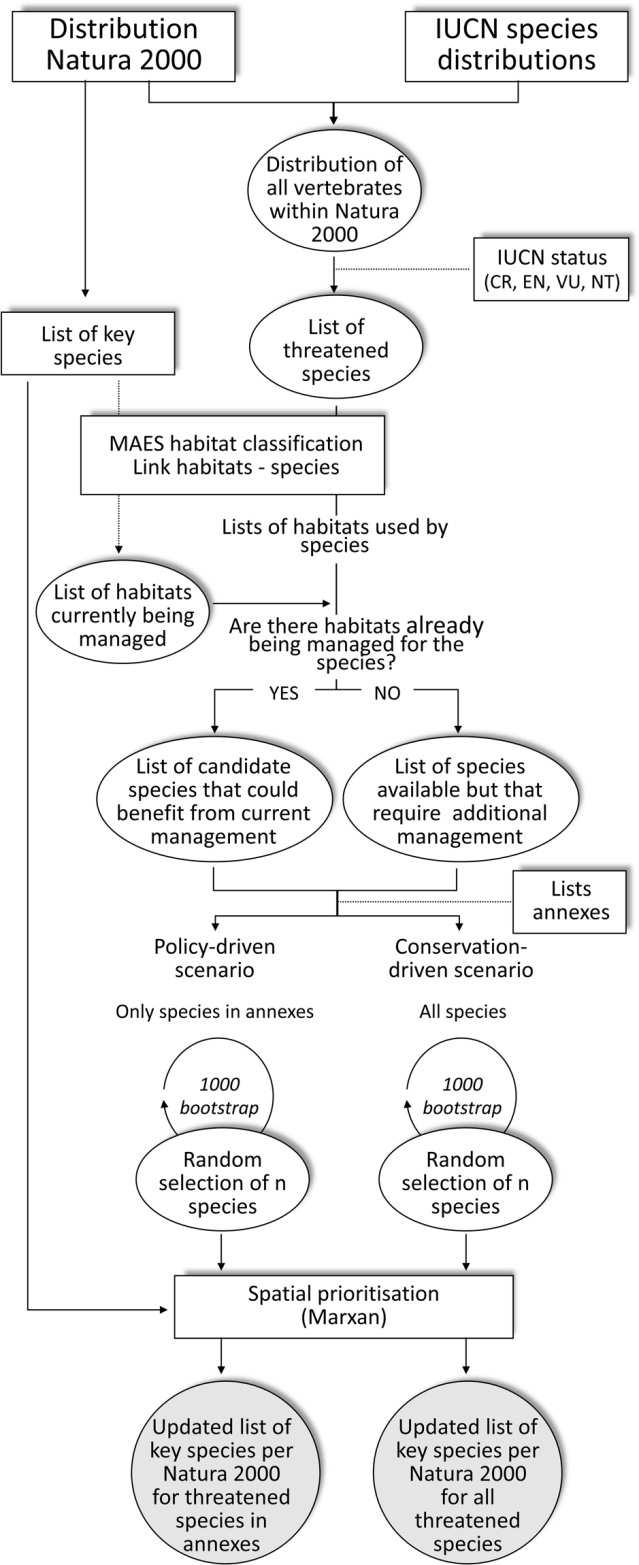
Flow chart of analyses carried out in this study. Rectangles indicate external data sources or software already available.

We sourced the most up to date extent of the Natura 2000 network from the European Environmental Agency (http://www.eea.europa.eu/data-and-maps/data/natura-8). This dataset contains the spatial distribution of 27,510 Natura 2000 sites and a list of target species for which each site was designated. These lists of target species represent the focus of conservation efforts within Natura 2000 sites, designated for protecting species in the Habitats and Birds Directives (sites can also be designated to protect habitats not covered in this study). However, they do not convey a full inventory neither of all species listed in the annexes of Directives nor of other species of conservation concern present in each Natura 2000 site. To gather a more comprehensive view of the value of the Natura 2000 sites for protecting species we compiled the occurrence data of all vertebrate species with potential presence across the whole network. For this demonstration exercise, we focused on vertebrate taxa given the availability of distribution data for all the species (sourced from^28^). We overlapped the IUCN distribution maps of vertebrates with the extent of the Natura 2000 sites to identify the sites with potential presence of vertebrates not considered in the lists of Natura 2000 target species (Fig. 3). To be conservative, only the areas where the species are native to (origin = ”native” according to the metadata in the IUCN dataset^16^) with a high certainty (presence = “extant”) were retained for the analyses.

### Gap analysis of Natura 2000

To assess the representativeness of Natura 2000 for the vertebrate species listed in the annexes of the Habitats and Birds Directives, we checked the total number of species that were cited as a target species in at least one Natura 2000 site. We then compared these species with those on the list of vertebrates included in the Directives’ annexes to identify potential gaps in the current network, summarised for each taxon independently. To explore more broadly the coverage of species in need of conservation action by the Directives, we also checked how many of the threatened species (Critically Endangered –CR-, Endangered –EN-, Vulnerable –VU-, and Near Threatened –NT- according to the most recent IUCN Red List database^16^) appeared on the list of Natura 2000 target species and on the list of potential gaps. We considered CR, EN, VU and NT species as these are the IUCN threat categories that approximately correspond to “unfavourable” status in the Directives^41^, and therefore, species for which management would be required^42^. This second analysis would give us an idea of the adequacy of the Directives at covering threatened species, as assessed by the IUCN criteria, and therefore of responding to those species in need of management action.

### Spatial prioritisation of upgrades to Natura 2000 lists of target species

We used the spatial planning software Marxan^43^ to identify priority areas for filling the representation gaps of threatened species within Natura 2000. We tested two alternative scenarios for upgrading the lists of target species in each Natura 2000 site by considering the addition of (1) threatened species listed in the Directives (policy-driven scenario) and (2) other threatened vertebrates, even if not listed in the Directives’ annexes (conservation-driven scenario). The latter is a more ambitious scenario, where we aimed to identify priority Natura 2000 sites where threatened species, not necessarily listed in the Directives, could be included as target species in a given Natura 2000 site and then become a management priority for the selected site. Within each of these two alternatives, Marxan optimises the allocation of threatened species to the existing lists of target species per Natura 2000 site by considering aspects like the representativeness, irreplaceability and complementarity of species across the network of protected areas. The outputs of these scenarios provide information on the relative importance of Natura 2000 sites in terms of conservation value for threatened species, and on the list of additional species that would be necessary to include under the list of target species of each Natura 2000 site to improve the representation of (1) either threatened species listed in the Directives that are not adequately represented today, or (2) the threatened vertebrates that are not listed in the Directives. None of these scenarios pose the designation of new Natura 2000 sites and they focus on identifying conservation opportunities to fill current management gaps with the resources already in place, whenever possible.

We also tried to make the most of current conservation efforts being carried out in Natura 2000 sites, and then minimise the impact of our recommendations in terms of additional conservation efforts required. With this aim, we used the list of target species of each Natura 2000 site as a surrogate of the current conservation efforts in place derived from the application of the Directives in that site. We assumed that habitats on which target species depend are being managed within each Natura 2000 site as part of the conservation efforts carried out for the maintenance of the species. This management effort could also benefit other threatened species that are also dependent on the same habitats. The association between each target species and their habitats was sourced from the linkages of species and habitat types to MAES ecosystems^44^. This document reports the types of habitats being used by each species. To be conservative and avoid overestimating types of habitats being managed in each Natura 2000 site, we kept only “preferred” and “suitable” habitats (*sensu*^44^), which represent the most important ecosystems for the species or where the species regularly occurs, respectively. To account for potential co-benefits of these management efforts for other species in our prioritisation exercise, we also identified habitat links for all the remaining vertebrates that occur within each Natura 2000 site and that are not declared as target species for that site (either species in the Directives or all threatened vertebrates, depending on the scenario being tested). This information was then used to identify a set of species that could be preferentially selected for upgrading the list of each Natura 2000 site from the list of all available species. Species with habitat requirements similar to other species already being managed in a given site were given priority in the analyses (Fig. 3). Whenever habitats currently managed in a given Natura 2000 site did not offer additional conservation opportunities to other species, we broadened the selection of species beyond these habitats. Therefore, the list of species made available during the optimisation process was the sum of all target species already being managed plus a number of additional species that occur in the Natura 2000 site and that occupy preferentially similar habitats to the existing managed target species. Given the strong North-South geographic gradient in the number of potential candidate species not declared as target across the Natura 2000 to be added to the network (Fig. 1b), not all candidate species were made available for a given site at the same time in the prioritisation process to avoid concentrating the efforts on a reduced number of sites. In particular, we constrained the addition of new species for each site to a given threshold (see below for more detail on thresholds). In sites where the number of candidate species was equal or lower than the set threshold, all species with occurrence in that site were potentially considered in the analysis to be added to the list of target species declared for that site. Whenever the number of candidate species was higher than the set threshold a random subset of these species was selected and made available for the prioritisation analysis, starting with species that already had habitats being managed and continuing whenever necessary with other species. This simulation was run 1000 times to account for the effect of stochastic selection of species, following the methodology proposed in^23^ (Fig. 3). We tested the effect of different thresholds by running independent analyses for 2, 5, 10, 20 and 50 species being allowed for selection. This sensitivity analysis resembles different planning strategies when trying to fill the representation gaps; setting low species thresholds represents a strategy seeking to disperse as much as possible conservation efforts across the EU, potentially at expenses of lower efficiency; on the other hand, higher thresholds resemble a strategy seeking to enhance efficiency, by concentrating efforts on a reduced number of sites, where large numbers of species could be co-managed.

For each of the 1000 simulations, we ran Marxan 100 times (5 million iterations each) and retained the best solution found for each simulation for further analyses^23^. The best solution among all 100 runs was identified as the one with the lowest score for the objective function being minimised (see below). We set a target of 10 for each species, meaning each of them would be represented in at least 10 Natura 2000 sites. Given the lack of consistent data on costs of management for different species we opted for using a constant cost for all planning units or Natura 2000 sites in our case (cost = 1). In addition, we did not use the boundary penalty available in Marxan’s objective function, given we were not interested in spatially clumping solutions. Therefore, our optimisation problem was:

1 $$\min \,{\sum }_{i=1}^{m}{x}_{i}{c}_{i}$$

2 $${\rm{S}}{\rm{u}}{\rm{b}}{\rm{j}}{\rm{e}}{\rm{c}}{\rm{t}}\,{\rm{t}}{\rm{o}}\,{\sum }_{(i=1)}^{m}{x}_{i}{a}_{i} > {t}_{j}{\rm{\forall }}j$$

where *x*_*i*_ is a control variable that takes a value of 1 when the Natura 2000 site *i* is selected and 0 otherwise; *c*_i_ is the cost of each *i*; *a*_i_ is the benefit for each species *j* provided by selecting site *i* (presence of species in a given Natura 2000 site in our case); and *t*_j_ is the target for each species. Under these premises the objective function that we tried to minimise was as stated in Eq. 3.

3 $$Objective\,function=\mathop{\sum }\limits_{i=1}^{n}{c}_{i}{x}_{i}+\mathop{\sum }\limits_{j=1}^{n}SP{F}_{j}H(s)(\frac{s}{{t}_{j}})$$

where there are *n* species under consideration; SPF_j_ is a Species Penalty Factor or weighting factor that applies for not achieving the desired representation target for each species *j*; H(s) is a Heaviside function that takes a value of 0 when *s*/*t*_j_ ≤ 0 and 1 otherwise; *s* is the shortfall in targets not achieved and is measured as *t*_j_-representation achieved (Eq. 2); the ratio *s*/*t*_j_ equals 1 when the species *j* is not represented within the solution and approaches 0 as the level of representation approaches the target amounts. We used a constant SPF = 10 for all species to ensure they all achieved the desired representation target^23^.

We then summarised the 1000 best solutions derived from 1000 subsets of species by calculating the frequency of occurrence of each Natura 2000 site across all best solutions and the frequency with which each species was selected in each Natura 2000 site. In this way, we obtained an estimate of the relative importance of each Natura 2000 site across Europe, regardless of the particular selection of species being tested, and the relevance of each Natura 2000 site for managing each species^23^. We also calculated the frequency with which each species was selected within a particular Natura 2000 site, being this an estimate of the relative importance of that site for the management of the particular species. In both scenarios, we also checked for the relationship between the local richness (number of species listed in the Directives or threatened species that occur within each Natura 2000 site) and the selection frequency of each site after 1000 bootstraps. We also checked for the relationship in the selection frequency between both scenarios, to see if Natura 2000 sites where consistently selected regardless the scenario being tested.

## Supplementary information

Supplementary figures
