# Supplementary material for: Realising the potential of Natura 2000 to achieve EU conservation goals as 2020 approaches
Source: Sci Rep. 2019 Nov 6;9:16087. doi: 10.1038/s41598-019-52625-4 (PMC6834658; doi:10.1038/s41598-019-52625-4)

Realising the potential of Natura 2000 to achieve EU conservation goals as 2020 approaches

Virgilio Hermoso, Alejandra Morán-Ordóñez, Stefano Canessa, Lluis Brotons

Supplementary Figure S1. Richness of species listed in the Directives (both threatened and not threatened). Natura 2000 sites are represented by their centroid for mapping purposes.

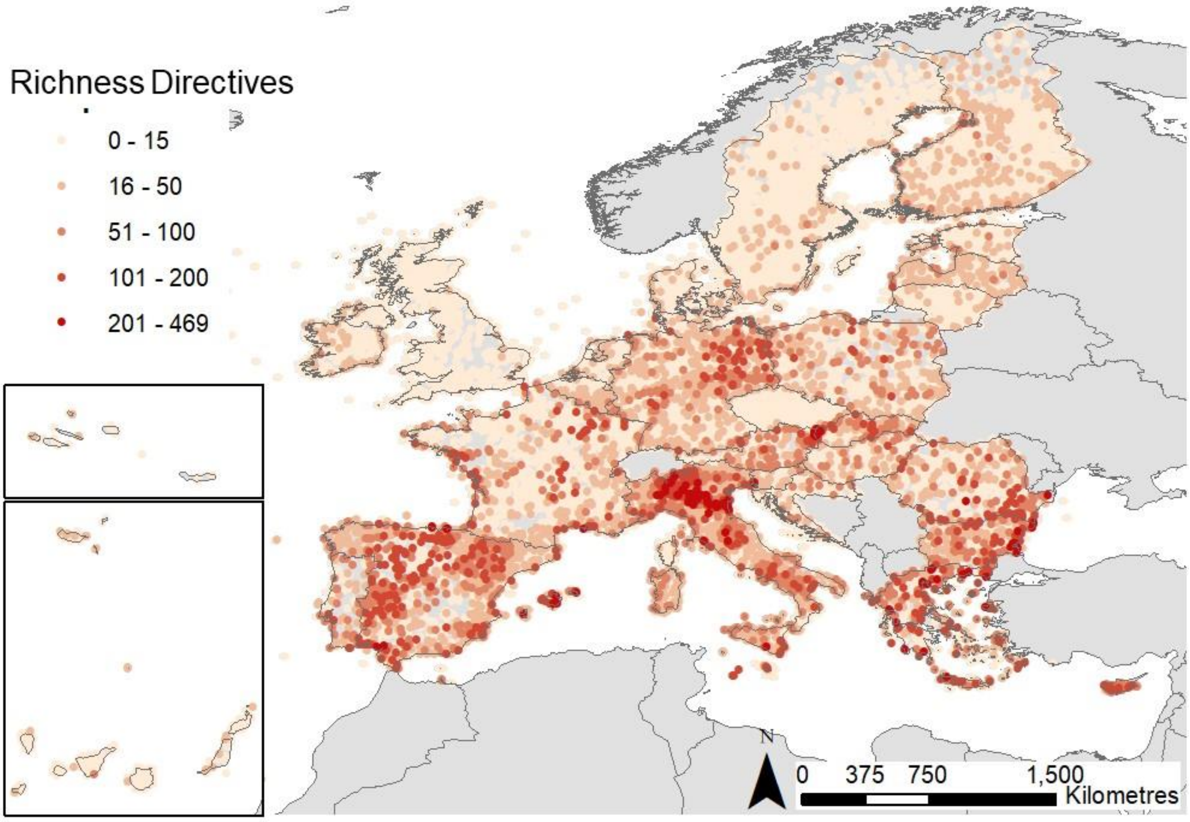

Supplementary Figure S2. Correlation between the frequency of selection of a particular N2000 site under a given scenario (policy-driven in top panel and conservation-driven in bottom panel) and its species richness.

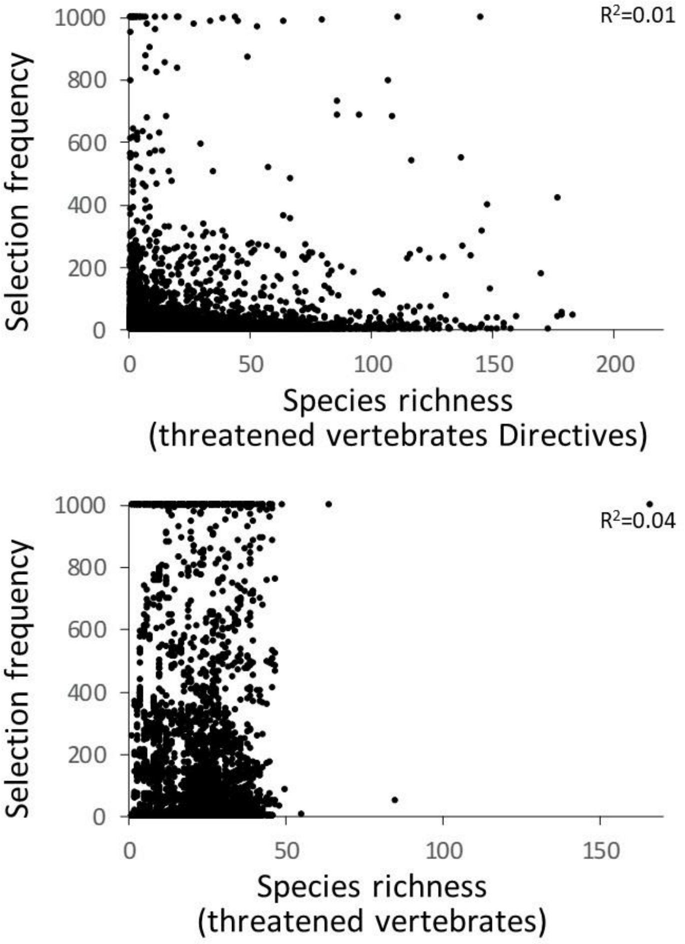

Supplementary Figure S3. Sensitivity of site selection frequency of Natura 2000 sites across different thresholds on the number of species allowed to be included in the upgraded lists of key species. Larger number of species allowed to be included in a single site led to a concentration of selection frequency in less sites.

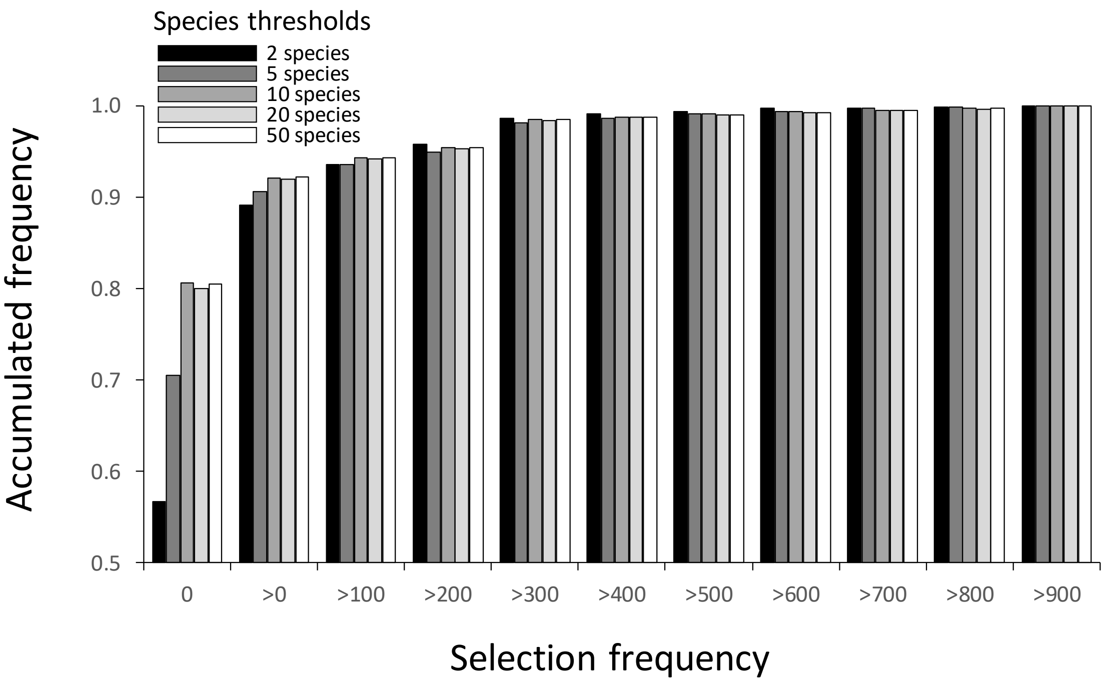

Supplementary Figure S4. Selection frequency of each Natura 2000 site under different thresholds of number of species allowed to be added to the lists of key species already into place for each Natura 2000 site, which are represented by their centroid for mapping purposes.

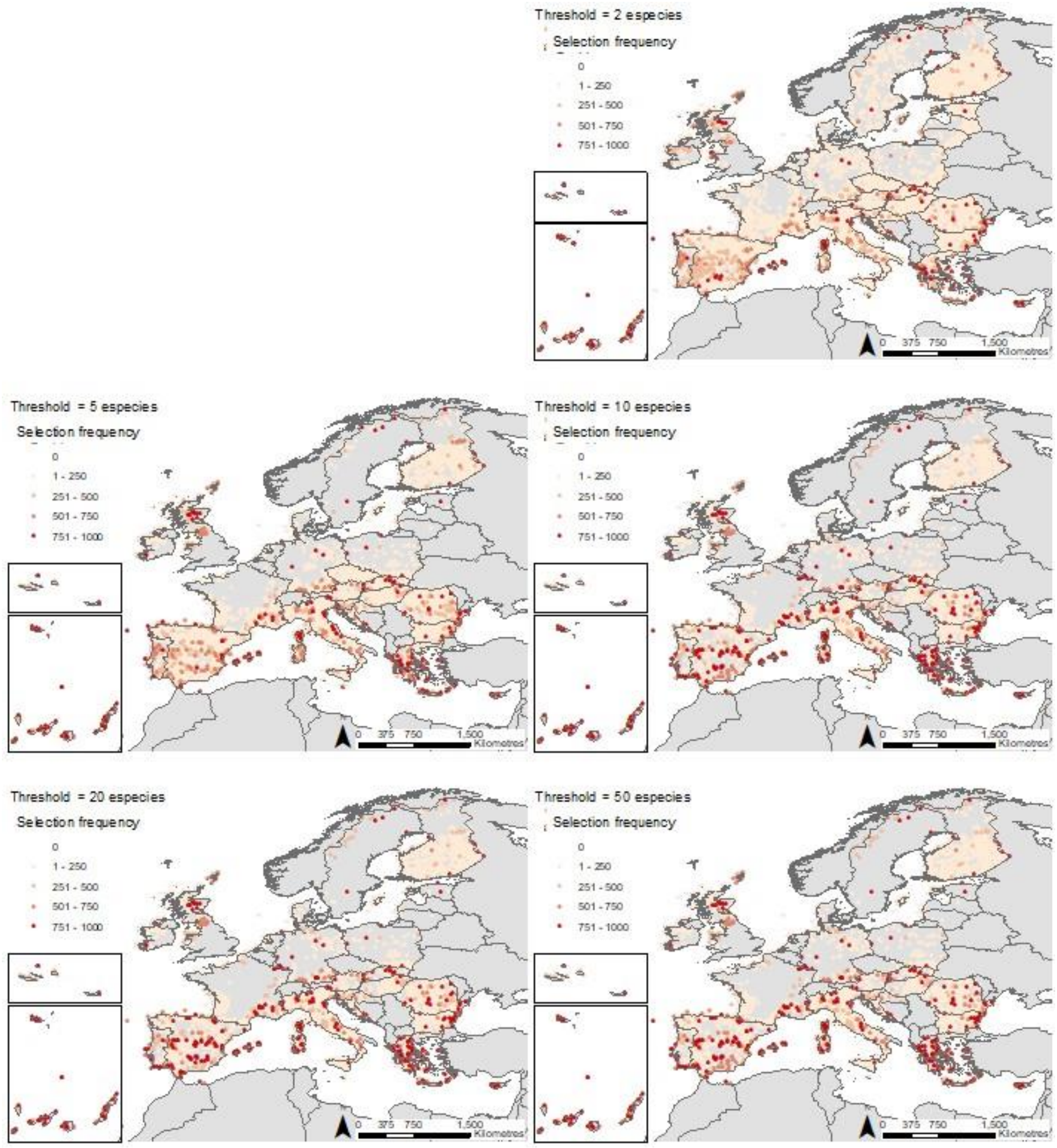

Supplement: Supplementary file 1 — Supplementary figures [file 41598_2019_52625_MOESM1_ESM.pdf]
